# *In vitro* synergistic interaction between *Melaleuca armillaris* essential oil and erythromycin against *Staphylococcus aureus* isolated from dairy cows

**DOI:** 10.3389/fvets.2022.1005616

**Published:** 2022-11-15

**Authors:** Daniel Buldain, Lihuel Gortari Castillo, Andrea Verónica Buchamer, Arnaldo Bandoni, Laura Marchetti, Nora Mestorino

**Affiliations:** ^1^Laboratorio de Estudios Farmacológicos y Toxicológicos (LEFyT), Facultad de Ciencias Veterinarias, Universidad Nacional de La Plata, La Plata, Argentina; ^2^Consejo Nacional de Investigaciones Científicas y Técnicas (CONICET), La Plata, Argentina; ^3^Facultad de Farmacia y Bioquímica, Universidad de Buenos Aires, Cátedra de Farmacognosia, Buenos Aires, Argentina; ^4^Instituto de Química y Metabolismo del Fármaco (IQUIMEFA), CONICET-Universidad de Buenos Aires, Buenos Aires, Argentina

**Keywords:** *Melaleuca armillaris*, essential oil, erythromycin, synergism, *Staphylococcus aureus*, mastitis

## Abstract

*Staphylococcus aureus* frequently causes subclinical mastitis around the world with a high impact on the milk industry and public health. Essential oils (EO) are recognized antimicrobials that can be synergistic with antibiotics. The main objective of this study was to evaluate the essential oil (EO) of *Melaleuca armillaris* as an adjuvant of erythromycin (ERY) for the alternative treatment of bovine mastitis caused by *S. aureus*. The Minimum Inhibitory and Bactericidal Concentrations (MIC and MBC) of EO, ERY, and its combinations were established against *S. aureus* at different pHs (7.4, 6.5 and 5.0), emulating extra and intracellular conditions. Sensitive (*N* = 3) and resistant (*N* = 3) strains to ERY and *S. aureus* ATCC 29213 as control were used. Math models were applied to describe the antibacterial activity of EO and combinations EO-ERY. The EO was bactericidal against all the strains independently of the pH with a slight improvement in acid conditions. The synergism between EO and ERY was estimated by the Fractional Inhibitory Concentration Index (FIC) and by mathematical modeling of the bacterial killing data. Synergism was observed with ERY, where combinations had bactericidal activity also even with pH modification. *M. armillaris* EO is an interesting adjuvant for ERY, being a promissory option for further analysis of intracellular efficacy against *S. aureus*.

## Introduction

Bovine mastitis is a pathology that commonly affects dairy cattle, being a contagious disease with a great impact on milk industry profitability (1). Sick animals must be treated appropriately to guarantee both their welfare and ability to produce high quality milk (2). However, using antimicrobials can present disadvantages such as partially low cure rates and the presence of residues in milk that could favor the emergence of resistant microorganisms. This implies the necessity to perform studies in finding alternative treatments (3). The innovative and alternative treatments may include reducing the use of antimicrobials, replacing them with more effective and safer treatments, or replacing their application with other types of compounds (2).

Staphylococci are frequently isolated in bovines with mastitis (4) and, particularly, *Staphylococcus aureus* has great relevance in this disease (5), being one of the main causative agents of intramammary infections in dairy cows worldwide (6). *S. aureus* can be found in the skin of the mammary gland and teat lesions, so the main reservoir is the infected udders, where the microorganisms adapt, survive, and grow (6). This microorganism can form biofilms, grow in cell cytoplasm, and cause persistent bacteraemia or chronic infection, or it can remain quiescent and reactivates months or years later. On the other hand, if the bacterial population density in the infectious focus is high, *S. aureus* can become resistant to most of the antibiotics used in monotherapy (7). Ideally antimicrobials used for the treatment of *S. aureus* infections should get into phagocytic cells and remain inside for an adequate time, not be metabolized in cells, have significant antimicrobial activity at acid pH, be able to be administered through the teat canal, and have good distribution in the mammary gland (8). Macrolides and β-lactams, among others, are antimicrobials commonly used for the treatment of bovine mastitis caused by *S. aureus* (9, 10).

Macrolides are considered bacteriostatic agents, although they can exert a bactericidal effect under certain conditions. These antimicrobials act on the 50S ribosomal subunit and interfere with protein synthesis, and they are particularly active against Gram-positive bacteria and mycoplasmas (11, 12). Macrolides have wide distribution in the body, prolonged elimination half-life, activity against important microbial pathogens. They can reach high intracellular concentrations (10) and decreases it antimicrobial activity at pH 5.0 (11, 13). Intracellular activity of most macrolides is low (14), it seems to be associated with inactivation due to acidic pH of the phagolysosome (pH near 5) where they are located (15). These antibiotics lose approximately 90% of their activity for each pH unit that decreases (16). However, new macrolides such as azithromycin present intracellular activity *in vivo* against *S. aureus* (13, 14).

Erythromycin (ERY) is a macrolide recommended for the treatment of bovine mastitis caused by Gram-positive cocci (9) and reach high concentrations in milk after parenteral or intramammary administration (17). It belongs to the group of antimicrobials classified as critically important for human medicine (18) and the European Medicines Agency (EMA) recommends avoiding irresponsible and unnecessary use in animal production (19).

Currently, the therapeutic tool available against bovine mastitis continues being the intramammary administration of antibiotics. However, this is associated with the problem of antimicrobial resistance, being necessary to seek new alternative treatment approaches. Plant antibacterial agents can act as important sources of new antibiotics and compounds targeting bacterial virulence, which can be used alone or in combination with existing drugs (20). Consequently, medicinal plants are becoming an excellent natural product resource for future antibacterial therapy. The use of phytotherapeutic resources is aimed to satisfy a market necessity, which prefers healthier products that guarantee less environmental impact and that allow us to face the problem of antimicrobial resistance to conventional therapeutic products. Combination therapy, which combines conventional antibiotics with natural products, represents a promising strategy to deal with antibiotic resistance in the future. There are numerous reports dating back to the use of medicinal plants in ethnoveterinary medicine for bovine mastitis treatment, however, it is essential to standardize the extracts and compounds used to guarantee their efficacy (20).

Essential oils (EOs) are mixtures of volatile compounds isolated from plants, and their main chemical components possess a wide range of potential bacterial inhibitors. They can act as bacteriostatic or bactericides. Their active molecules have a great variety of target sites, mainly in the plasma membrane and in the cytoplasm, and in certain situations, they completely alter the morphology of cells. Effects of EOs generally lead to destabilization of the phospholipid bilayer, destruction of plasma membrane function and composition, loss of vital intracellular components, and inactivation of enzyme mechanisms (21). On the other hand, EOs have the potential to act as synergistic adjuvants of antibiotics, decreasing the concentration of antimicrobials to inhibit a microorganism (22–25).

*Melaleuca armillaris* EO had antimicrobial activity against *S. aureus* isolated from cows when it was applied alone (26, 27) and in combination with antibiotics like cloxacillin (28) and rifaximin (29). Therefore, the objective of this study was to combine this EO with ERY evaluating their interaction against *S. aureus* at different pH emulating pH conditions at the extra and intracellular level.

## Materials and methods

### *Melaleuca armillaris* essential oil extraction and characterization

The EO was obtained by steam distillation of fresh leaves and herbaceous branches of *M. armillaris* plants from the surroundings of Coronel Brandsen town (latitude 35°06′18.9″S and longitude 58°10′57.0″W), Buenos Aires, Argentina. A sample of the *M. armillaris* plants used was deposited in the LPAG Herbarium of the Faculty of Agrarian and Forestry Sciences, UNLP (30). As was described in previous works (27–29), the EO was dried with sodium sulfate anhydrous, filtered with a cotton funnel, and stored at 4°C in an amber glass bottle. EO composition was characterized by gas chromatography combined with mass spectrometry and flame ionization detection (GC-MS-FID; Agilent, Agilent Technologies, Santa Clara, CA, USA) (28), and the physicochemical characteristics were determined also (27).

### *Staphylococcus aureus* strains and susceptibility against ERY

*S. aureus* ATCC 29213 reference strain and six wild-type *S. aureus* strains, isolated according to the National Mastitis Council procedure (31), from subclinical mastitis Holstein cows were used. Sampling was carried out following the Guide for the Care and Use of Agricultural Animals in Agricultural Research and Teaching (32) and was approved by the Institutional Committee (CICUAL) of the Faculty of Veterinary Sciences, National University of La Plata (47.3.15J). Strains were identified phenotypically as a Gram-positive, coagulase-positive, catalase-positive, β-hemolytic, Voges Proskauer-positive, fermentation of trehalose, mannitol, and maltose. The susceptibility was checked by disk diffusion test using erythromycin disk 15 μg disk (Britania, Argentina) and *S. aureus* ATCC 25923 as quality control (33). Also we evaluated susceptibility to penicillin (10 IU), oxacillin (1 μg), clindamycin (2 μg), rifampin (5 μg), ciprofloxacin (5 μg) from Oxoid, England; cefoxitin (30 μg), gentamicin (10 μg), tetracycline (30 μg), azithromycin (15 μg), cefoperazone (30 μg), cephalexin (30 μg), enrofloxacin (5 μg), vancomycin (30 μg) from Britania, Argentina.

### Minimum inhibitory and bactericidal concentration (MIC and MBC) of ERY and EO

The MIC of EO and ERY was performed by microdilution in broth using 96-well polystyrene microtiter plates with Mueller Hinton broth (MHB) (Biokar Diagnostics, France). A 0.5% of Tween 80 (Biopack, Argentina) was added to enhance the EO dissolution. The broth pH was adjusted to 7.4, 6.5 and 5.0 by addition of hydrochloric acid 1N (Anedra, Argentina), emulating pH conditions of extracellular and intracellular level sites. The ERY (Parafarm, Argentina) range of concentrations analyzed (applying a scheme of two-fold serial dilution) were between 1024 and 0.007 μg/mL. EO concentrations tested were between 50 and 0.1 μL/mL. In both cases each well was inoculated with a final bacterial concentration of 5 × 10^5^ CFU/mL. Microplates were incubated at 35°C for 18–24 h. MIC was established as the lowest concentration which inhibits the bacterial growth. Each determination was done in triplicate. Positive and negative controls contained MHB with Tween 80 (0.5%) were included in the test.

The MBC was determined by inoculation spreading of 25 μL from each well showing no evident bacterial growth (after establishing the MIC) in nutritive agar plates for colony counting after incubation at 35°C for 18–24 h. The MBC was established as the first antimicrobial concentration which produce the fall of 99.9% from the initial inoculum.

### Antimicrobial activity of combinations ERY/EO

The MIC of ERY-EO combinations at pH 7.4, 6.5 and 5.0 was established by the checkerboard technique (34) against the same strains used before. Thus, presence or absence of synergism was analyzed.

The design of the microtiter plate consisted of a row with two-fold serial dilution of EO and a column with two-fold serial dilution of ERY (antimicrobials MIC control). The intermediate wells had ERY/EO combinations in different proportions. The bacterial inoculums of *S. aureus* were 5 × 10^5^ CFU/mL per well and the incubation was carried out at 35°C for 18–24 h. The MIC was established as the combination that inhibit the bacterial growth.

The results interpretation was similar for the MIC of individual antimicrobials but considering it as a mix. The fractional inhibitory concentration index (FIC) was determined by the following equation:


$$\begin{array}{l} FIC=\frac{\left(A\right)}{\left(MIC\right)a}+\frac{\left(B\right)}{\left(MIC\right)b} \end{array}$$

A synergistic effect exists if FIC ≤ 0.5, partial synergism (PS) if 0.5 < FIC < 1, indifference or addition (I) if 1 ≤ FIC < 2, and antagonism (A) if FIC ≥ 2 (34, 35). Also, the same formula was applied to evaluate the synergism in terms of MBC, considering the same cutoff.

### Time-kill assay and antibacterial activity index of ERY, EO and combinations ERY-EO

Time kill-assays for the 3 *S. aureus* strains resistant to ERY, exposing them to different amounts of the antimicrobial alone, and combined with EO at pH 7.4, 6.5 and 5.0 were performed. The concentrations were selected based on the MIC obtained in each case. For EO-ERY combinations 0.5 MIC, 1 MIC, 2 MIC, 4 MIC and 8 MIC were used. In the case of ERY alone 0.5 MIC, 1 MIC, 2 MIC and 4 MIC at pH 7.4 and 0.5 MIC, 1 MIC and 2 MIC at pH 6.5 and 5.0, respectively, were assayed. The reason of this was the high MIC value (particularly in acid conditions) for these strains making difficult the solubilization of the antibiotic in the culture media.

A tube for each condition to be evaluated containing a volume of 1 mL including Mueller-Hinton Broth with 0.5% Tween 80 (pH 7.4, 6.5, and 5.0), antimicrobial, and a bacterial concentration of 5 × 10^5^ CFU/mL was prepared. Also, a positive (without antimicrobial) and a negative (without antimicrobial and inoculums) control were included. Tubes were incubated at 35°C and the bacterial plate count was performed sampling at 0, 2, 4, 8, 12, and 24 h after incubating at 35°C by 24 h. The experiment was performed in triplicate for each strain. With the data obtained Log_10_ (CFU/mL) *vs*. time graphs were constructed and the antibacterial activity index (*E*) was evaluated. *E* index was defined as the difference in Log_10_ between the bacterial count (CFU/mL) at the initial time (nt-0) and at the end of the assay (nt-24): *E* = (nt-24) – (nt-0). Three theoretical breakpoints to establish the bacteriostatic effect (*E* = 0), bactericidal effect (*E* = −3), and effect of virtual eradication of bacteria (*E* = −4) (36).

Then the *E* index *vs*. ERY concentration was graphed to compare what happens in presence and absence of EO. Wild type resistant strains were grouped according to the MIC, obtaining two groups of three strains for each one (resistant and sensitive), using the mean of triplicates for each strain.

### Math modeling of *E* index

The *E* index values *vs*. ERY concentration data was mathematically modeled using a sigmoid model similar to a maximum response model (27, 37, 38). In this way we could analyzed deeply the effect of EO in the antimicrobial activity of ERY against *S. aureus* under the different conditions evaluated. The model equation was:


$$\begin{array}{l} E=E_{0}-\;\left(E_{max}.C^{\gamma }\right)/\left(C_{50}^{\gamma }+\;C^{\gamma }\right) \end{array}$$

Where *E* is the index *E* (Log_10_ CFU/mL) for a concentration C (μg/mL), *E*_0_ is the index *E* in the absence of the antimicrobial (Log_10_ CFU/mL), *E*_max_ is the maximum reduction in Log_10_ of *E*_0_, C_50_ (μg/mL) is the concentration that causes 50% of the reduction of the *E*_max_, and γ is the coefficient of sigmoidicity. The experimental data were fitted with the nonlinear least squares regression model using Sigma Plot software (Sigma Plot 12.0, 2011).

The C_50_ of ERY was compared at the 3 pHs by using the one-way analysis of variance (ANOVA) and the Tukey-Kramer multiple comparison test with a level of significance established at *p* < 0.05. On the other hand, the C_50_ of ERY was compared with the same parameter obtained in the combinations with EO by means of the t test for unpaired data with a level of significance established at *p* < 0.05.

## Results

We worked with 3 wild type strains sensitive to ERY (SA13, SA96 and SA139), 3 wild type strains resistant to ERY (SA78A, SA79A and SA86B), and the reference strain ATCC 29213. Isolates SA78A, SA79A, and SA86B were also resistant to penicillin, azithromycin, and clindamycin. MICs and MBCs of ERY for all strains are shown in Table 1. We found that MIC of ERY was very low against *S. aureus* sensitive strains compared to resistant ones (0.5 and 1,024 μg/mL, respectively). In acid conditions the antibiotic was less effective, suffering a substantial loss of potency. This was particularly evident by the increment of MIC up to 2 times at pH 6.5, and 16 times at pH 5.0 compared to the MIC at pH of 7.4 for sensitive strains. Regarding resistant isolates, the loss of potency led to MIC values higher than 1,024 μg/μL. The MBC/MIC ratio for sensitive strains at pH 7.4 was about of 64, 256 at pH 6.5 and at pH 5.0 it was >32 (since the MBC was established as >256 μg/mL). For ERY resistant strains, the MBC was established as >1,024 μg/mL, showing an increase respect to MIC at pH 7.4. At pH 5.0 these values were also determined as >1,024 μg/mL. In this way, the extreme loss of potency suffered by this antibiotic in acidic media is clearly confirmed.

**Table 1:** MIC and MBC values of ERY for all the strains evaluated at pH 7.4, 6.5, and 5.0.

	**pH 7.4**		**pH 6.5**		**pH 5.0**	
	**MIC**	**MBC**	**MIC**	**MBC**	**MIC**	**MBC**
**Strain**	**ERY**	**ERY**	**ERY**	**ERY**	**ERY**	**ERY**
	**μg/mL**	**μg/mL**	**μg/mL**	**μg/mL**	**μg/mL**	**μg/mL**
ATCC 29213	0.5	32	1	> 256	8	>256
SA13	0.5	32	1	256	8	>256
SA96	0.5	32	1	256	8	>256
SA139	0.5	32	1	256	8	>256
SA78A	1,024	>1,024	>1,024	>1,024	>1,024	>1,024
SA79A	1,024	>1,024	>1,024	>1,024	>1,024	>1,024
SA86B	1,024	>1,024	>1,024	>1,024	>1,024	>1,024

In the case of EO, MIC and MBC (shown in Table 2) were slightly lower with the media acidity for all strains. The ratio MBC/MIC was 2 for the reference and sensitive wild type strains independently of the pH. For resistant wild type strains, this relation was 4 at pH 7.4 and the double at pH 6.5 and 5.0.

**Table 2:** MIC and MBC values of EO for all the strains evaluated at pH 7.4, 6.5, and 5.0.

**Strain**	**pH 7.4**		**pH 6.5**		**pH 5.0**	
	**MIC**	**MBC**	**MIC**	**MBC**	**MIC**	**MBC**
	**EO**	**EO**	**EO**	**EO**	**EO**	**EO**
	**μL/mL**	**μL/mL**	**μL/mL**	**μL/mL**	**μL/mL**	**μL/mL**
ATCC 29213	25	50	25	50	12.5	25
SA 13	12.5	25	12.5	25	6.25	12.5
SA 96	12.5	25	12.5	25	6.25	12.5
SA 139	12.5	25	12.5	25	6.25	12.5
SA 78A	12.5	50	6.25	50	3.1	25
SA 79A	12.5	50	6.25	50	3.1	25
SA 86B	12.5	50	6.25	50	3.1	25

In Table 3 are listed the combinations of ERY and EO which presented lower FIC values. It is possible to observe synergism and partial synergism, depending on the strain and pH condition. The decrease of ERY concentration necessary to produce bacteria inhibition was important for resistant strains in presence of EO.

**Table 3:** Results of synergism tests of EO/ERY combinations at pH 7.4, 6.5, and 5.0 in terms of MIC (FIC evaluation).

**Strain**	**pH 7.4**		**pH 6.5**		**pH 5.0**	
	**MIC**	**FIC**	**MIC**	**FIC**	**MIC**	**FIC**
	**EO/ERY**		**EO/ERY**		**EO/ERY**	
	**(μL/mL)/(μg/mL)**		**(μL/mL)/(μg/mL)**		**(μL/mL)/(μg/mL)**	
ATCC 29213	12.5/0.125	0.75	3.1/0.25	0.37	3.1/2	0.5
SA 13	6.25/0.125	0.75	3.1/0.25	0.5	3.1/2	0.75
SA 96	6.25/0.125	0.75	3.1/0.25	0.5	3.1/2	0.75
SA 139	6.25/0.125	0.75	3.1/0.25	0.5	3.1/2	0.75
SA 78A	6.25/64	0.56	3.1/128	<0.62	1.5/128	<0.62
SA 79A	6.25/64	0.56	3.1/128	<0.62	1.5/128	<0.62
SA 86B	6.25/64	0.56	3.1/128	<0.62	1.5/128	<0.62

We also evaluated the synergism considering a similar index that FIC but in terms of the bactericidal combinations found. So, in Table 4 we show the EO/ERY combinations which had bactericidal activity and the respective fractional bactericidal concentration index (FBC). With these results, we also can mention that the combination EO/ERY has a synergic or partially synergic effect depending on the strain or pH condition, but it is more relevant concerning the analysis of inhibitory concentrations. At pH 7.4 the 3 resistant isolates presented FIC values of 0.56, in which the amount of antibiotic necessary for inhibition decreased 16 times (1,024 vs. 64 μg/mL) in the presence of 6.25 μL/mL of EO (MIC of EO decreased by half). At pH 6.5, the FIC coefficient was < 0.65 (we could not establish it exactly since the MIC of the antibiotic only in this condition was determined as >1,024 μg/mL). In this case, the MIC of the antibiotic decreased at least 8 times (>1,024 to 128 μg/mL) but in the presence of 3.1 μL/mL of the essence, that is, the EO decreased its MIC by half. Finally, at pH 5.0 the situation was very similar to that observed at pH 6.5, since the MIC of ERY was reduced from >1,024 to 128 μg/mL, with the difference that the amount of plant extract was even less. The concentration of EO in the mixture at this pH was 1.5 μL/mL. Something similar was observed with the FBC index, but the synergic effects seem to be more important analyzing the bactericidal effect respect the inhibition.

**Table 4:** Results of synergism tests of EO/ERY combinations at pH 7.4, 6.5, and 5.0 in terms of MBC.

**Strain**	**pH 7.4**		**pH 6.5**		**pH 5.0**	
	**MBC** **EO/ERY** **(μL/mL)/(μg/mL)**	**FBC**	**MBC** **EO/ERY** **(μL/mL)/(μg/mL)**	**FBC**	**MBC** **EO/ERY** **(μL/mL)/(μg/mL)**	**FBC**
ATCC 29213	6.25/0.5	0.14	6.25/2	<0.13	12.5/4	<0.52
SA 13	3.1/1	0.16	6.25/2	0.26	6.25/4	<0.52
SA 96	3.1/1	0.16	3.1/4	0.13	6.25/4	<0.52
SA 139	3.1/1	0.16	6.25/2	0.26	6.25/8	<0.53
SA 78A	25/64	<0.56	25/64	<0.56	12.5/64	<0.56
SA 79A	25/64	<0.56	25/64	<0.56	12.5/64	<0.56
SA 86B	25/64	<0.56	25/128	<0.63	12.5/64	<0.56

**Figure 1:**
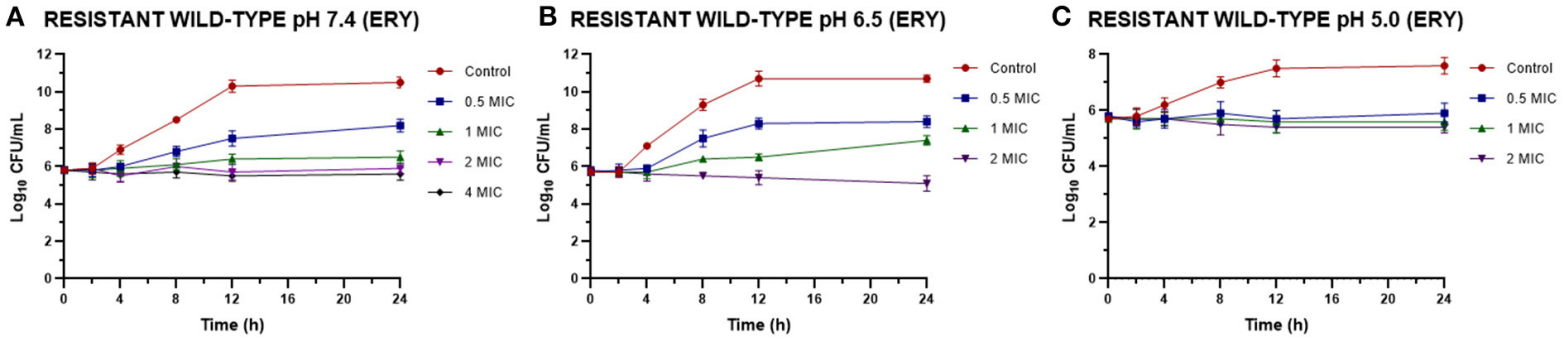
Time-kill curves for ERY against *S. aureus* resistant strains (*n* = 3, using the mean of triplicates for each strain) at pH 7.4 **(A)**, 6.5 **(B)**, and 5.0 **(C)** [minimum inhibitory concentration (MIC) = 1,024 μg/mL at pH 7.4 and 2,048 μg/mL at pH 6.5 and 5.0].

Once the MIC of ERY alone and combined with EO was established we perform time-kill assays for resistant strains. Figure 1 shows the effect of different ERY concentrations at pH 7.4, 6.5 and 5.0, where even at high concentrations the bacteriostatic effect prevails. Analyzing the bacterial death curves for *S. aureus* (resistant strains) against ERY (Figure 1), it can be observed again how, at the concentrations evaluated, the antimicrobial acts in a bacteriostatic way, since after 24 h of contact it was not possible to obtain a significant drop in the bacterial count, regardless of the pH and the sensitivity profile of the strains. The incidence of pH is observed in the amount of antibiotic necessary to achieve the same effect, since the concentrations in all cases increase with the acidification of the culture medium.

**Figure 2:**
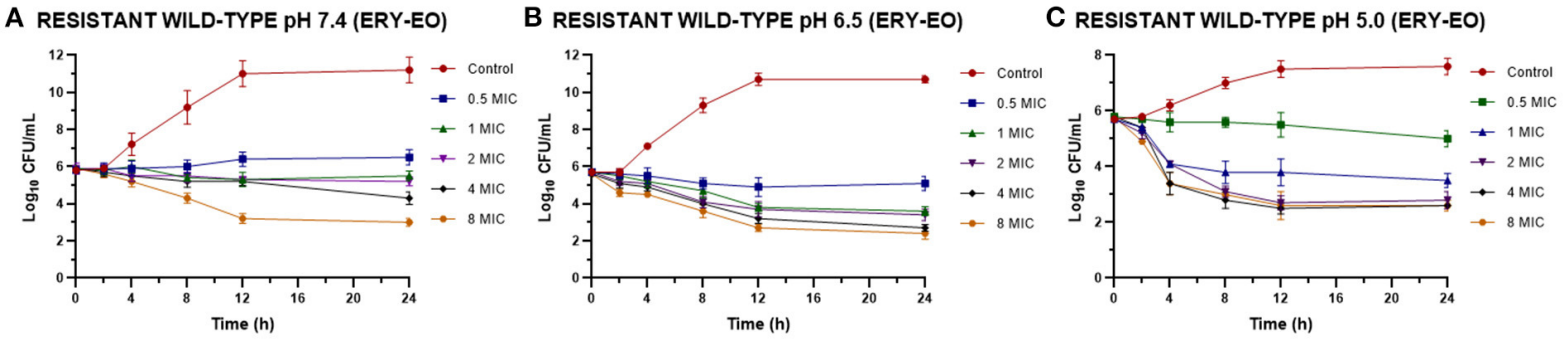
Time-kill curves for ERY/EO combinations against *S. aureus* resistant strains (*n* = 3, using the mean of triplicates for each strain) at pH 7.4 **(A)**, 6.5 **(B)**, and 5.0 **(C)** [minimum inhibitory concentration (MIC) = 64 μg/mL at pH 7.4 and 128 μg/mL at pH 6.5 and 5.0].

However, the addition of EO allow a drop in the bacterial count during the time evaluated, even with lower ERY amounts (Figure 2).

**Figure 3:**
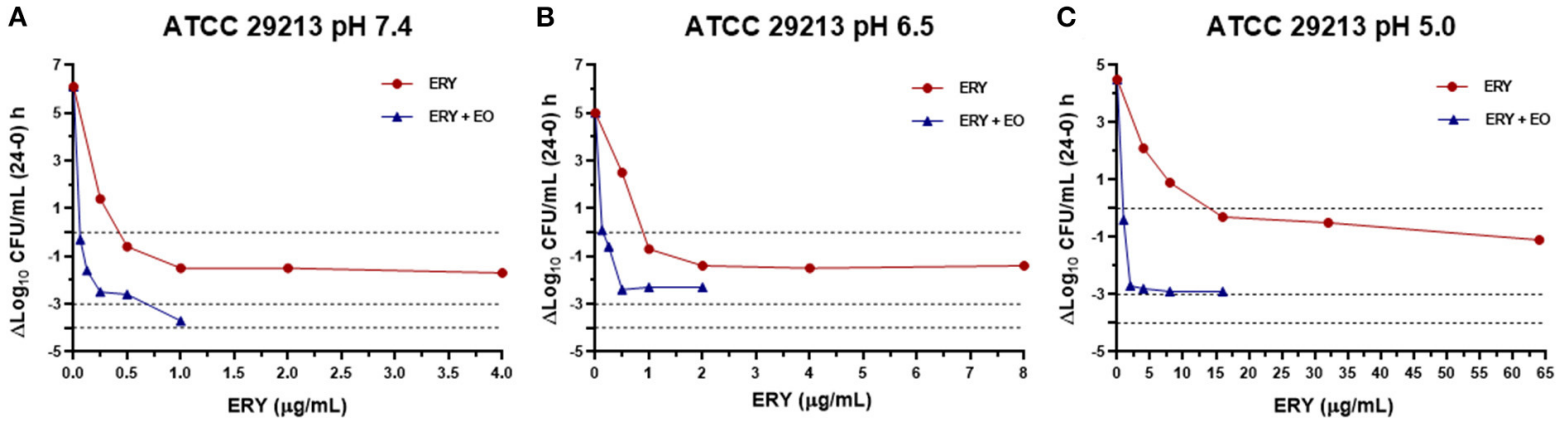
Graphic representation of the antibacterial effect (E: ΔLog_10_ CFU/mL 24–0 h) of ERY alone and combined with EO against *S. aureus* ATCC 29213 at pH 7.4 **(A)**, 6.5 **(B)**, and 5.0 **(C)**.

**Figure 4:**
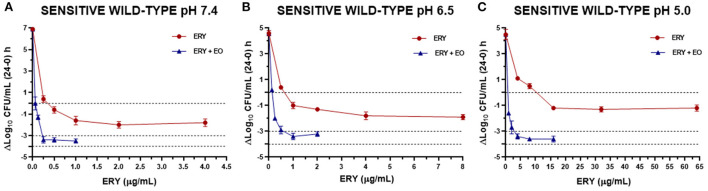
Graphic representation of the antibacterial effect (E: ΔLog_10_ CFU/mL 24–0 h) of ERY alone and combined with EO against *S. aureus* sensitive strains grouped (using the mean for each strain) at pH 7.4 **(A)**, 6.5 **(B)**, and 5.0 **(C)**.

**Figure 5:**
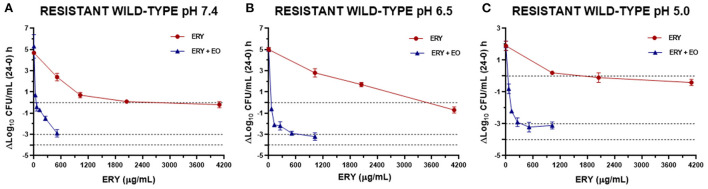
Graphic representation of the antibacterial effect (E: ΔLog_10_ CFU/mL 24–0 h) of ERY alone and combined with EO against *S. aureus* resistant strains grouped (using the mean for each strain) at pH 7.4 **(A)**, 6.5 **(B)**, and 5.0 **(C)**.

The antibacterial index *E* was graphed vs. ERY concentrations at pH 7.4, 6.5 and 5.0 for the reference strain (Figure 3), sensitive strains (Figure 4) and resistant strains (Figure 5). In these figures is possible to observe again the synergic effect of the addition of EO in ERY activity, also considering the change of pH.

The antibacterial effect *E vs*. ERY concentration curves at the 3 pHs evaluated were mathematically modeled. The parameters obtained for ERY alone are shown in Table 5 and the same parameters for the combination with EO are described in Table 6. For the reference strain and isolates sensitive to this macrolide, it was possible to optimally adjust the model with R close to 1 in all the evaluated conditions. On the other hand, when evaluating resistant strains using only ERY, the data corresponding to pH 6.5 and 5.0 did not show good adjustments (R close to 0). This is probably because of a lower number of points in the curve due 4 and 8 MIC were not evaluated for dissolution problems. For all strains confronted with ERY, it is notorious how the C_50_ increases with the decrease in pH, the increase at pH 5.0 being very significant (*p* < 0.001). In turn, the presence of EO allows the C_50_ of ERY to be significantly lower than when used alone at the 3 pHs evaluated (*p* < 0.0001). This parameter at pH 7.4 decreases from 543.167 to 189.503 μg/mL with 12.5 μL/mL of EO. For resistant isolates with 4 MIC at pH 6.5 and 2 MIC at pH 5.0, bactericidal effects are achieved. While at pH 7.4 a greater amount of both is required to achieve this effect. An explanation for this fact could be that ERY resistant strains appear to be more susceptible to pH lower than 7.4, where *E*_max_ is lower for the control without antimicrobials of resistant strains compared to the sensitive and reference strains.

**Table 5:** Parameters estimated by applying the sigmoid model to evaluate the antibacterial activity index for *S. aureus* at pH 7.4, 6.5, and 5.0 vs. erythromycin.

**ERY**	**ATCC 29213**			**Sensitive strains**			**Resistant strains**		
pH	7.4	6.5	5.0	7.4	6.5	5.0	7.4	6.5	5.0
R	1.00	1.00	0.99	0.99	0.99	0.99	1.00	–	–
*E*_max_ (Log_10_ CFU/mL)	7.78	6.47	5.72	9.05	6.54	5.94	4.87	–	–
⋎	2.15	3.47	1.25	1.17	1.47	1.28	2.38	–	–
C_50_ (μg/mL)	0.21	0.57	5.16	0.12^a^	0.32^a^	3.85^b^	543.17	–	–
*E*_0_ (Log_10_ CFU/mL)	6.11	5.02	4.48	6.87	4.59	4.33	4.67	–	–

Sigmoid model. E_0_, E-index without erythromycin; E_max_, maximum reduction in bacterial count; C_50_, concentration of erythromycin necessary to achieve 50% of the maximum antibacterial effect; ⋎, sigmoidicity coefficient. Wild strains were grouped by minimal inhibitory concentration (MIC) similarity. For the strains 78A, 79A, and 86B at pH 6.5 and 5.0 it was not possible to carry out the modeling, the model yielded R adjustment values close to zero. Different lowercase letters between the pHs mean statistical differences (p < 0.01). Values without significant differences have the similar letter (a, b).

**Table 6:** Parameters estimated by applying the sigmoid model to evaluate the antibacterial activity index for *S. aureus* at pH 7.4, 6.5, and 5.0 vs. erythromycin in the presence of EO.

**EO-ERY**	**ATCC 29213**			**Sensitive strains**			**Resistant strains**		
pH	7.4	6.5	5.0	7.4	6.5	5.0	7.4	6.5	5.0
R	0.99	0.99	1.00	0.99	0.99	0.99	0.99	0.99	1.00
*E*_max_ (Log_10_ CFU/mL)	10.69	7.62	7.38	10.79	7.94	8.08	13.85	8.26	5.12
⋎	0.64	1.35	3.89	1.27	1.99	1.61	0.39	1.19	1.87
C_50_ (μg/mL) of ERY	0.03	0.08	0.84	0.04^a^	0.11^a^	0.53^b^	189.50^c^	33.59^d^	61.15^e^
*E*_0_ (Log_10_ CFU/mL)	6.11	5.01	4.48	6.87	4.59	4.34	5.34	4.97	1.89

Sigmoid model. E_0_, E index in the absence of antimicrobials; E_max_, maximum reduction in bacterial count; C_50_, concentration of erythromycin (in the presence of EO) necessary to achieve 50% of the maximum antibacterial effect; ⋎, sigmoidicity coefficient. Wild strains were grouped by minimal inhibitory concentration (MIC) similarity. Different lowercase letters between the pHs mean statistical differences (p < 0.05). Values without significant differences have the similar letter (a, b, c, d, e).

## Discussion

Our results shows that ERY acts in a bacteriostatic way and the *M. armillaris* EO is bactericidal, since if this ratio is >4 it is indicative that a compound is bacteriostatic and bactericidal if it is lower than 4 (39).

The combination of *M. armillaris* EO and ERY was interesting, particularly in the case of wild *S. aureus* strains resistant to this macrolide. We previously demonstrate the EO antimicrobial activity against *S. aureus* and the efficacy as adjuvant of cloxacillin (28) and rifaximin (29). The strains resistant to ERY evaluated in this work (SA78A, SA79A and SA86B) had very high MIC values (1,024 μg/mL), losing antimicrobial activity in acid conditions as was mentioned before. The combination with EO allowed to obtain mixtures whose FIC was close to 0.5, establishing partial synergism. Small amounts of *M. armillaris* EO were able to decrease significantly the concentration required to inhibit these *S. aureus* strains with high resistance to ERY.

There are few studies about synergism of ERY and plant extracts. Synergism against *S. aureus* has been found when combining this antibiotic with the *Lippia alba* EO (40) and the extract of *Indigofera suffruticosa* (41). Magi et al. (42) found that ERY is synergistic with carvacrol (terpene found in some essential oils) against Streptococci.

There are several resistance mechanisms developed by *S. aureus* that affect the activity of macrolides (11, 43). In first place, due to the modification of the target site by methylation or mutation, preventing the binding of the antibiotic to its ribosomal target. A second mechanism involves the efflux of the antibiotic, and a third the inactivation of the drug. Modification of the ribosomal target confers broad-spectrum resistance to macrolides while efflux and inactivation affect only some of these molecules (44).

It is difficult to establish the mechanism by which *M. armillaris* EO and ERY exerts a synergistic effect. The large number of compounds found in the extract could have effect on different bacterial targets. The EO composition was previously described (28). The 1.8-cineole (main component found in the *M. armillaris* EO) can disintegrate the cell membrane and reducing the cytoplasm, causing damage to the structure of *S. aureus* (45). Regarding the other components of this EO, it has been postulated that α-Pinene, Terpinen-4-ol, sabinene, β-Myrcene and α-Terpinene would also be involved in the interaction with the cell membrane, where they dissolve in the phospholipid bilayer aligning between fatty acid chains. This physical distortion of the structure would cause expansion and destabilization of the membrane, increasing its fluidity, which in turn would increase passive permeability (46). A possible explanation for the synergy between the EO and ERY would be that destabilization in the membrane, cell wall and an eventual decrease in the activity of efflux pumps would increase the arrival of antibiotics into the bacterial cell interior, therefore it would become even more concentrated and facilitate interaction with the site of action at the ribosomal level.

Piatkoswka et al. (43), who studied strains of *S. aureus* resistant to ERY, said that resistance was the consequence of a strong decrease in the permeability of the cell wall to ERY. According to these authors, this variant of resistance mechanisms turns out to be the most efficient, creating the most resistant strains, with a MIC value >1,024 μg/mL. The highly resistant strains did not present a large accumulation of the macrolide at intracellular level by destabilizing the cell membrane, so the barrier that stops their entry would be in the bacterial wall. Among other behaviors observed, cells from highly resistant strains tended to form larger and more stable aggregates, indicating that they differ in cell wall composition from less resistant ones. It is then possible that the activity of EO has implications on the cell wall.

An important aspect that presented differences was the change in bacterial death curves shapes. All resistant strains exposed to ERY alone at the 3 pH conditions presented growth curves that correspond to a bacteriostatic antibiotic, which was also reflected in that the MBC/MIC ratios were in all cases >4 (as mentioned above this is common for bacteriostatic antimicrobials).

With the addition of EO to the culture medium, bactericidal effects were obtained. This was also reflected in the E-index analysis. Like was observed for other classes of antibiotics like cloxacillin (28) and rifaximin (29), the E-Index *vs*. antibiotic concentration, show how the curve is shifted toward lower concentrations of ERY in the presence of EO. On the other hand, it is clearly stating how the addition of the plant extract favors the scope of the bactericidal effect. Again, the acidification of the culture medium presents an effect like that described above with the analysis of MIC values for the antibiotic.

Using the sigmoid model similar to the maximum response model to adjust the antibacterial index data as a function of ERY concentration, it is possible to observe what happens with the maximum effect (*E*_max_) and the necessary concentration (C_50_) to achieve 50% of *E*_max_. There are no publications that report mathematical modeling of the antimicrobial activity of natural products combined with antibiotics. We have previously published the mathematical modeling of the activity of the *M. armillaris* EO on *S. aureus* (27). In this work we establish the usefulness of this type of models to compare the addition of adjuvants in the activity of antimicrobials. It is clear how the C_50_ of erythromycin decreases because of EO addition, reinforcing the results of synergism observed by previously analyzing the FIC and FBC indices.

Considering that macrolides can concentrate at the intracellular level, mainly within macrophages and polymorphonuclear leukocytes (47), the MIC and MBC of ERY combined with EO could be reached at the subcellular level. It has been reported that ERY can accumulate between 4 and 38 times more at the intracellular level than in the extracellular environment in macrophages, 8 times in polymorphonuclear neutrophils and 6 to 12 times in epithelial cells (48, 49). Therefore, it is feasible to reach these levels, transforming its combination with *M. armillaris* EO into a good alternative to evaluate for the treatment of *S. aureus* at the intracellular level.

There are few *in vivo* studies with EOs, Byung-Wook et al. (50) treated clinical mastitis in cows with *Origanum vulgare* EO, resulting in a decrease in *S. aureus* infection without causing swelling, redness, pain, and increased temperature in the udder. There are some EO-based products on the market for intramammary application, such as Phyto-Mast. This is recommended for intramammary use in lactation and drying. Thyme (*Thymus vulgaris*) EO is the antimicrobial active component and when used in cattle it did not present any irritating and inflammatory effect (51). Regarding residues in milk (taking thymol as marker), they were only detected 12 h after intramammary administration to goats (52) and cows (53). These findings allow us to consider the feasibility of administering EOs intramammary in the future as part of the treatment against mastitis. It is interesting to take advantage of the secondary metabolites produced by plants with pharmacological potential in the control of bovine mastitis in the context of both the problem of bacterial resistance and in the search for organic productions free of chemical residues. However, it is essential to standardize the extracts to ensure the quality and efficacy of the formulations.

## Conclusions

The *M. armillaris* EO was synergic with erythromycin. The MICs and MBCs decreased with the addition of small amounts of EO, for both sensitive and resistant strains. Erythromycin had bacteriostatic activity when using alone, but when it was combined with the EO it behaved as a bactericidal antibiotic. If we consider that erythromycin can accumulate intracellularly, the bactericidal effect achieved with the EO combination would be considered as a promising alternative for the treatment of staphylococcal infections in bovine mastitis in a future, taking this work as starting point. The analysis of biological systems using mathematical models allows to obtain more information that simplifies collecting data from the observation of the results of an *in vitro* test. We will continue investigating about the intracellular efficacy of the combination between this macrolide and the EO.

## Data availability statement

The original contributions presented in the study are included in the article/supplementary material, further inquiries can be directed to the corresponding author/s.

## Author contributions

NM: conceived, supervised, and designed the experiments. DB: performed all the experimental assay, statistical analysis, and written the manuscript. LGC, AVB, and LM: contributed with experimental assays. AB: performed the EO quality assay. All authors contributed to the review, revision, and approved the final manuscript.

## Funding

This work is partially financed by the Laboratory of Pharmacological and Toxicological Studies (LEFyT) and the National Agency for Scientific and Technical Promotion (ANPCyT) (PICT 2018-00932 and PICT 2020-01429).

## Conflict of interest

The authors declare that the research was conducted in the absence of any commercial or financial relationships that could be construed as a potential conflict of interest.

## Publisher's note

All claims expressed in this article are solely those of the authors and do not necessarily represent those of their affiliated organizations, or those of the publisher, the editors and the reviewers. Any product that may be evaluated in this article, or claim that may be made by its manufacturer, is not guaranteed or endorsed by the publisher.
